# Proteomic architecture of frailty across the spectrum of cardiovascular disease

**DOI:** 10.1111/acel.13978

**Published:** 2023-09-20

**Authors:** Andrew S. Perry, Shilin Zhao, Priya Gajjar, Venkatesh L. Murthy, Benoit Lehallier, Patricia Miller, Sangeeta Nair, Colin Neill, J. Jeffrey Carr, William Fearon, Samir Kapadia, Dharam Kumbhani, Linda Gillam, JoAnn Lindenfeld, Laurie Farrell, Megan M. Marron, Qu Tian, Anne B. Newman, Joanne Murabito, Robert E. Gerszten, Matthew Nayor, Sammy Elmariah, Brian R. Lindman, Ravi Shah

**Affiliations:** ^1^ Vanderbilt Translational and Clinical Cardiovascular Research Center Vanderbilt University School of Medicine Nashville Tennessee USA; ^2^ Cardiovascular Medicine Section, Department of Medicine Boston University School of Medicine Boston Massachusetts USA; ^3^ Department of Medicine University of Michigan Ann Arbor Michigan USA; ^4^ Alkahest, Inc. San Carlos California USA; ^5^ Department of Medicine, and Department of Biostatistics Boston University School of Medicine Boston Massachusetts USA; ^6^ Department of Medicine, Division of Cardiovascular Medicine University of Wisconsin Hospital and Clinics Madison Wisconsin USA; ^7^ Department of Medicine, Division of Cardiology Stanford Medical Center Palo Alto California USA; ^8^ Department of Medicine, Division of Cardiology Cleveland Clinic Foundation Cleveland Ohio USA; ^9^ Department of Medicine, Division of Cardiology University of Texas Southwestern Medical Center Dallas Texas USA; ^10^ Department of Cardiovascular Medicine Morristown Medical Center Morristown New Jersey USA; ^11^ Broad Institute of Harvard and MIT Cambridge Massachusetts USA; ^12^ Department of Epidemiology, Graduate School of Public Health University of Pittsburgh Pittsburgh Pennsylvania USA; ^13^ National Institute on Aging, National Institutes of Health Baltimore Maryland USA; ^14^ Departments of Medicine and Clinical and Translational Science University of Pittsburgh Pittsburgh Pennsylvania USA; ^15^ Sections of Cardiovascular Medicine and Preventive Medicine and Epidemiology, Department of Medicine Boston University School of Medicine Boston Massachusetts USA; ^16^ Cardiovascular Institute, Beth Israel Deaconess Medical Center, Harvard Medical School Boston Massachusetts USA; ^17^ Department of Medicine, Division of Cardiology The University of California San Francisco California USA

**Keywords:** cardiovascular disease, frailty, proteomics

## Abstract

While frailty is a prominent risk factor in an aging population, the underlying biology of frailty is incompletely described. Here, we integrate 979 circulating proteins across a wide range of physiologies with 12 measures of frailty in a prospective discovery cohort of 809 individuals with severe aortic stenosis (AS) undergoing transcatheter aortic valve implantation. Our aim was to characterize the proteomic architecture of frailty in a highly susceptible population and study its relation to clinical outcome and systems‐wide phenotypes to define potential novel, clinically relevant frailty biology. Proteomic signatures (specifically of physical function) were related to post‐intervention outcome in AS, specifying pathways of innate immunity, cell growth/senescence, fibrosis/metabolism, and a host of proteins not widely described in human aging. In published cohorts, the “frailty proteome” displayed heterogeneous trajectories across age (20–100 years, age only explaining a small fraction of variance) and were associated with cardiac and non‐cardiac phenotypes and outcomes across two broad validation cohorts (*N* > 35,000) over ≈2–3 decades. These findings suggest the importance of precision biomarkers of underlying multi‐organ health status in age‐related morbidity and frailty.

## Graphical abstract

Our study involved observational cohort studies to identify circulating, proteomic markers of frailty and examine the relation of the “frailty proteome” with multimorbidity and long‐term outcomes.

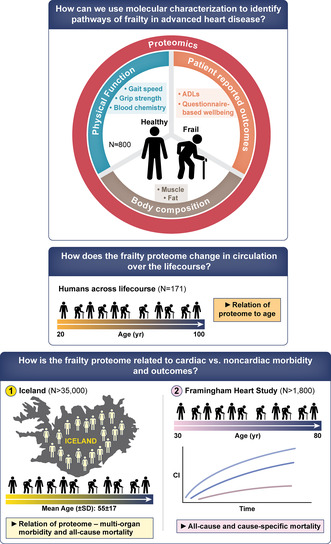

**Abbreviations**
ADLs: activities of daily living
AS: aortic stenosis
BMI: body mass index
CVD: cardiovascular disease
eGFR: estimated glomerular filtration rate
EQ‐VAS: EuroQol visual analog scale
FDR: false discovery rate
FHS: Framingham Heart Study
HDL: high density lipoprotein
KCCQ‐12: Kansas City Cardiomyopathy Questionnaire Summary Score
LASSO: least absolute shrinkage and selection operator
LOESS: locally estimated scatterplot smoothing
MNA‐SF: Mini Nutritional Assessment Short Form
NT‐proBNP: N‐terminal pro‐brain natriuretic peptide
PCA: principal component analysis
PHQ‐2: Patient Health Questionnaire‐2
TAVI: transcatheter aortic valve implantation

## 1 INTRODUCTION

With improvements in cardiac intervention and prevention during the past three decades, individuals who would have previously succumbed to acute, non‐communicable diseases (cardiovascular disease [CVD], oncologic) now survive to an older age with multiple advanced chronic conditions (Ijaz et al., 2022). This changing clinical landscape challenges the routine application of high‐risk therapy in higher risk individuals specifically in age‐related conditions, like CVD (Jha et al., 2017; Leon et al., 2010; Smith et al., 2011; Reardon et al., 2017; Waksman et al., 2018), where an interplay between cardiac *and* non‐cardiac physiology impact outcomes. In this context, understanding how frailty—an impaired ability to maintain homeostasis during physiologic stress (Clegg et al., 2013)—modifies treatment response is critical. Despite associations of several frailty measures with clinical outcomes (Clegg et al., 2013; Guralnik et al., 1994, 1995; Ijaz et al., 2022), there remains significant heterogeneity in how frailty is assessed among older adults, including those with CVD (Rohrmann, 2020), with concerns around how best to reproducibly define and quantify frailty across centers and conditions as major limitations to widespread adoption (Rockwood & Howlett, 2018). While efforts to define molecular correlates of chronological aging abound (Ahadi et al., 2020; Basisty et al., 2020; Emilsson et al., 2018; Lehallier et al., 2019, 2020; Sebastiani et al., 2021; Tanaka et al., 2018), their application in tissues accessible clinically (e.g., blood) has largely been limited to an epidemiologic context (Landino et al., 2021; Liu et al., 2022; Sathyan et al., 2020; Tanaka et al., 2020), without a clear ability to define the impact of circulating biochemistry on downstream, post‐therapy outcome (Ferrucci & Fabbri, 2018; Ramonfaur et al., 2022). Given the potential for early identification of “accelerated” aging and molecular intervention (Sinha et al., 2014), identifying pathways of human frailty related to poorer tolerance of intervention may prioritize adjunctive avenues of therapy and investigation to enhance resilience in this growing population.

Here, we hypothesized that biological pathways of frailty—revealed through integrating 12 measures of frailty with comprehensive proteomic profiling—would identify older individuals at high risk of mortality despite intervention. We studied 809 individuals with symptomatic, severe aortic stenosis (AS) undergoing transcatheter valve implantation (TAVI)—an age‐related cardiovascular condition in which frailty has had prognostic implication (Kiani et al., 2020). We quantified 979 circulating proteins alongside 12 measures of frailty encompassing body composition, cognition, nutrition, patient‐centered assessment of well‐being, functional measures, and biochemistry. We developed, validated, and characterized proteomic signatures of three composite axes of frailty against post‐TAVI mortality, and explored the generalizability of our findings and their age dependence across multiple studies (35,559 community‐dwelling adults from Iceland (Ferkingstad et al., 2021); human studies across the life‐course (Lehallier et al., 2019); and 1894 community‐dwelling individuals in the Framingham Heart Study [FHS]). Ultimately, we sought to define a proteomic architecture of frailty in structural heart disease and characterize its broad relevance to multi‐organ phenotypes, function, and outcome to inform future studies of risk and therapy.

## 2 RESULTS

### 2.1 Study populations

To derive proteomic correlates of frailty in advanced heart disease, we studied 809 individuals with severe AS from a multicenter prospective cohort study (Perry et al., 2022; Stein et al., 2022) where frailty measures were systematically collected, split into two samples: (1) a derivation sample (*N* = 233) that had complete data on 12 measures of frailty and (2) a validation sample (*N* = 576) comprised of the remainder of our multicenter AS cohort that did not have complete data on the 12 frailty measures (Table 1). Both samples had follow‐up for vital status. Overall, the AS cohort had a median age 83 years (range 46–100 years, 44% women), with a high prevalence of coronary artery disease (70%) and diabetes (nearly 40%). The derivation and validation samples were largely comparable, with some imbalance in diabetes prevalence (43% in validation vs. 28% in derivation), body mass index, and in some measures of self‐reported health status (e.g., KCCQ‐12 and EQ‐VAS).

**TABLE 1:** Baseline characteristics of the aortic stenosis cohort.

Characteristic	Overall (*N* = 809)	Derivation (*N* = 233)	Validation (*N* = 576)	*p*‐value
Age	83 (77, 88)	84 (78, 87)	83 (76, 88)	0.3
Female	352 (44%)	106 (45%)	246 (43%)	0.5
Race				
White	781 (97%)	226 (97%)	555 (96%)	0.1
Black	18 (2.2%)	3 (1.3%)	15 (2.6%)	
Asian	8 (1.0%)	2 (0.9%)	6 (1.0%)	
Other	2 (0.2%)	2 (0.9%)	0 (0%)	
Body mass index (kg/m^2^)	27.6 (24.3, 31.9)	26.8 (23.7, 30.1)	28.1 (24.6, 32.5)	0.004
History of smoking	421 (52%); 0.5%	130 (56%); 0.9%	291 (51%); 0.3%	0.2
Coronary artery disease	565 (70%)	157 (67%)	408 (71%)	0.3
Diabetes mellitus	315 (39%); 0.1%	66 (28%)	249 (43%); 0.2%	<0.001
Katz Index of Independence in ADLs				
1	5 (0.7%); 6.3%	1 (0.4%)	4 (0.8%); 8.9%	0.3
2	13 (1.7%); 6.3%	2 (0.9%)	11 (2.1%); 8.9%	
3	9 (1.2%); 6.3%	1 (0.4%)	8 (1.5%); 8.9%	
4	34 (4.5%); 6.3%	6 (2.6%)	28 (5.3%); 8.9%	
5	121 (16%); 6.3%	36 (15%)	85 (16%); 8.9%	
6	576 (76%); 6.3%	187 (80%)	389 (74%); 8.9%	
KCCQ‐12 summary score	47 (30, 66); 6.1%	53 (36, 70)	43 (27, 61); 8.5%	<0.001
EQ‐VAS score	60 (40, 75); 6.7%	60 (50, 80)	50 (40, 75); 9.4%	<0.001
PHQ‐2				
0	357 (47%); 5.2%	116 (50%); 0%	241 (45%); 7.3%	0.8
1	124 (16%); 5.2%	38 (16%); 0%	86 (16%); 7.3%	
2	138 (18%); 5.2%	40 (17%); 0%	98 (18%); 7.3%	
3	65 (8.5%); 5.2%	18 (7.7%); 0%	47 (8.8%); 7.3%	
4	42 (5.5%); 5.2%	10 (4.3%); 0%	32 (6.0%); 7.3%	
5	16 (2.1%); 5.2%	6 (2.6%); 0%	10 (1.9%); 7.3%	
6	25 (3.3%); 5.2%	5 (2.1%); 0%	20 (3.7%); 7.3%	
Nutrition (MNA‐SF)	12 (10, 13); 8.0%	12 (10, 13)	11 (10, 12); 11%	0.08
Mini‐Cog total score				
0	20 (2.7%); 6.9%	8 (3.4%)	12 (2.3%); 9.7%	>0.9
1	87 (12%); 6.9%	27 (12%)	60 (12%); 9.7%	
2	133 (18%); 6.9%	43 (18%)	90 (17%); 9.7%	
3	157 (21%); 6.9%	45 (19%)	112 (22%); 9.7%	
4	172 (23%); 6.9%	53 (23%)	119 (23%); 9.7%	
5	184 (24%); 6.9%	57 (24%)	127 (24%); 9.7%	
Average gait speed (m/s)	0.68 (0.49, 0.86); 8.4%	0.68 (0.54, 0.86)	0.67 (0.48, 0.87); 12%	0.6
Average handgrip strength (kg)	20 (14, 27); 9.9%	18 (13, 26)	20 (14, 27); 14%	0.4
Psoas muscle area index (cm/m^2^)	6.74 (5.62, 8.04); 54%	6.60 (5.63, 7.89)	7.03 (5.62, 8.29); 76%	0.3
Visceral fat area index (cm/m^2^)	69 (43, 96); 63%	67 (43, 91)	70 (43, 104); 88%	0.6
Albumin (g/dL)	3.80 (3.40, 4.10); 0.6%	3.70 (3.40, 4.00)	3.80 (3.40, 4.20); 0.9%	0.05
Hemoglobin (mg/dL)	12.30 (11.00, 13.50); 0.6%	12.60 (11.30, 13.80)	12.20 (10.90, 13.30); 0.9%	0.004
eGFR	58 (45, 75); 0.6%	63 (48, 77); 0.9%	56 (43, 73); 0.5%	0.03

*Note*: Continuous variables are reported as median (25th percentile, 75th percentile); % missing (if any). Categorical variables are reported as *N* (%); % missing (if any). *P* values are from Wilcoxon rank sum test for continuous variables and chi‐squared test for categorical variables where expected cell counts were >5. Fisher's exact test was used for all other categorical variables.

Abbreviations: ADLs, Activities of Daily Living; KCCQ‐12, Kansas City Cardiomyopathy Questionnaire summary score; EQ‐VAS, EuroQol Visual Analog Scale; PHQ‐2, Patient Health Questionnaire‐2; MNA‐SF, Mini Nutritional Assessment Short Form; eGFR, estimated glomerular filtration rate.

In general, our replication cohorts had broad age distribution (Icelandic studies, *N* = 35,559, mean age 55 ± 17 years; 57% women; Eiriksdottir et al., 2021; Ferkingstad et al., 2021); multicenter study of 171 individuals across four centers in the United States and Europe, age range 21–107 years; 51% women (Lehallier et al., 2019); FHS cohort, *N* = 1894 age 55 ± 10, 54% women, Table S2 (Liu et al., 2022). The FHS cohort had lower prevalent cardiometabolic morbidity in FHS relative to our AS cohort, consistent with its younger mean age and being a community‐based population. As we conducted original analyses in FHS, the study population is reported in Table S2. For detailed cohort characteristics of the other replication cohorts, the reader is directed to the parent publications (Eiriksdottir et al., 2021; Ferkingstad et al., 2021; Lehallier et al., 2019).

### 2.2 Multidimensional frailty measures are classified into three broad phenotypic groups

Distribution of frailty measures in our derivation sample is in Table 1 with correlations in Figure S1. Given the physiologic and statistical relatedness across frailty measures, we used principal component analysis (PCA) to identify composite axes of frailty (Figure 2). The top three principal components (PCs) explained ≈49% of variance in the frailty phenome studied (loadings for each of the three PCs in Figure 2a). The first PC (“axis”) was weighted predominantly on patient‐reported metrics of well‐being, including PHQ‐2, EQ‐VAS, KCCQ‐12, and MNA‐SF (hereafter called “patient‐reported outcomes”). The second axis was weighted highly on body composition (visceral fat area index, psoas muscle area index) with lesser weights for grip strength and cognitive scores (hereafter labeled “body composition”). The third axis was weighted on objective measures of physical function (Katz ADL score, gait speed, grip strength) and biochemical measures included in frailty (hemoglobin, albumin), and was termed “physical function.” Frailty axes demonstrated a similar heterogeneity across age as previously reported for individual frailty metrics (Rohrmann, 2020; Figure 2b), with only a modest correlation between each component with age (maximum Spearman |*ρ*| = 0.20). In addition, consistent with known phenotypic dimorphism by sex, we observed higher body composition and physical functional scores for men relative to women (Figure S3).

### 2.3 Proteomic correlates of frailty identify older adults at high risk for mortality after cardiac intervention

To identify proteomic signatures of frailty, we next used linear regression methods (both ordinary and LASSO) across the proteome as independent variables with each individual frailty measure or each composite frailty axis (from the PCA above) as the dependent variable in separate models (results in the Data File S1). Hemoglobin and albumin were related to the greatest number of proteins, followed by gait speed, nutrition, and KCCQ‐12. LASSO regressions for each of the three frailty axes selected 191 unique proteins, with fewer proteins selected in models for patient‐reported outcomes than for body composition or physical function. LASSO‐based protein signatures of each frailty axis (protein “score” for that phenotypic axis, see Section 4) had variable model fits, with model fits generally poorest for patient‐reported outcomes (fit for hold‐out folds during LASSO optimization shown in Figure S4a; fit across entire derivation sample shown in Figure S4b). To validate these protein scores of frailty, we imputed missing frailty data in the validation sample (using multivariate imputation by chained equations, see Section 4) to correlate frailty axes with the protein scores. This demonstrated similar relations as the derivation sample: a poor relation in models for patient‐reported outcomes (Spearman *ρ* = 0.17), moderate correlations for body composition (Spearman *ρ* = 0.40), and physical function (Spearman *ρ* = 0.41). Given the need for complete data in PCA, the use of imputation for data missingness was restricted only to test replication of association of protein scores to the composite axes of frailty. We did not observe effect modification by sex on the relationship between individual proteins and frailty axes after FDR adjustment (Benjamini–Hochberg) for multiple testing of interaction terms. Each protein score was related to the frailty measures most heavily loaded in the parent frailty axis from which it was derived (Figure S5). Accordingly, each protein score exhibited a similar age and sex relation as the parent frailty axes (maximum Spearman |*ρ*| = 0.29 for age across all protein scores).

We next assessed the relation of each protein score and frailty axis from which it was derived with all‐cause mortality. Across a median 3.2 years of follow‐up (in derivation sample; 25th‐75th percentile 1.3–3.6 years), each of the three frailty axes had point estimates for post‐TAVI mortality in a protective range, with only physical function significantly related to mortality after clinical risk adjustment (Figure 3). Protein scores of frailty exhibited similar estimates for mortality in both derivation and validation samples, generally robust to multivariable adjustment at a median 2.9 years follow‐up (25th‐75th percentile 1.2–3.9 years). Of note, in sensitivity analyses, associations with mortality were robust to adjustment for simpler biomarkers canonically associated with cardiovascular mortality (NT‐proBNP, hemoglobin, albumin (Ibrahim & Januzzi Jr., 2018; Table S3).

### 2.4 The proteome implicates both known and novel pathways of human frailty

We used proteins associated with the 12 frailty measures in linear models for pathway analysis, respectively (at a 5% FDR). The proteins identified implicated broad pathways of innate and adaptive immunity (e.g., cytokine signaling and TNF), canonical cell growth and signaling pathways (e.g., PI3K‐Akt signaling), and organ fibrosis and metabolism (e.g., extracellular matrix remodeling and turnover, glycosylation; Figure S6). In addition to proteins with known relation to body composition (e.g., leptin and insulin‐like growth factor binding proteins), several novel proteins with roles in adipose tissue metabolism and inflammation were identified (in association with frailty axes), including PLIN1 (higher expression related to increased adipocyte size, altered lipid handling, and improved whole‐body glucose tolerance Kern et al., 2004), INHBC (involved in activin C signaling in adipocytes via ALK7, implicated in human obesity; Carlsson et al., 2009; Goebel et al., 2022), MEP1B (metalloproteinase; murine deletion results in weight gain with limited human data; Png et al., 2023), ADGRG1 (implicated in islet function and reduced islet expression in diabetes and hyperglycemic stress (Duner et al., 2016), and RET (implicated in anorectic responses downstream of GDF‐15; Li et al., 2017). Similarly, the physical function proteome (proteins associated with “physical function” frailty axis) included proteins previously widely implicated in inflammation, muscle function, and cachexia, including GDF‐15 (Crunkhorn, 2020; Siddiqui et al., 2022), MSTN (Schafer et al., 2016), IL6 (Strassmann et al., 1992), and FABP4 (Kim et al., 2013; Lee et al., 2017), as well as a host of proteins not widely reported in frailty biology, with roles in innate inflammation (IL‐17A Ying et al., 2022), IL‐10 Deans et al., 2009, TLR3 Graber et al., 2018), myogenesis or muscle regeneration (ITGA11 Grassot et al., 2014, EFNA1 Alonso‐Martin et al., 2016, LRRN1 McKellar et al., 2021), cachexia (ASGR1 Narasimhan et al., 2020), lysosomal metabolism (dynamic with muscle atrophy; cathepsin CTSL; Wu et al., 2011), organ fibrosis (MZB1 Schiller et al., 2017), oxidative stress (LGALS9 Nunoue et al., 2021), neurogenesis (SDC1 Mouthon et al., 2020), and metabolism (ANGPTL4 Gusarova et al., 2018, PLIN1).

### 2.5 Chronological age does not fully account for broad variability in the frailty proteome

Given relevance of implicated pathways across the life‐course (e.g., immunity, cell growth, and metabolism), we next sought to quantify the extent to which proteins related to frailty axes were explained by age. In the AS cohort, age only accounted for a small fraction of the total variability in protein scores (Figure 4a), with sex, and BMI accounting for more of the variability, and protein scores were weakly related to age (Figure 4b). To test whether this observation was present in a broader age range, we examined 50 circulating proteins that overlapped with proteins associated with any frailty axis (from single protein linear regression) in 171 individuals across four cohorts (age 21–107 years; Lehallier et al., 2019; Figure 4c), resolving three predominant patterns with age: (1) proteins exhibiting higher (GDF‐15, IGFBP2, REN, consistent with prior studies; Liu et al., 2021; van den Beld et al., 2019) or (2) lower (CA6, MSTN, RET) circulating levels at older age; and (3) proteins that did not exhibit a clear monotonic association with age (LEP, LTBR, IL4R, EPO; characteristic raw data plots in Figure S7). These life‐course patterns may largely have been established by the time advanced heart disease (AS) requiring intervention had developed (purple line demonstrating age range of AS cohort, Figure 4c), accounting for the low variation explained by age in our AS sample.

### 2.6 The frailty proteome and clinical risk

Given the physiologic relevance of frailty‐implicated pathways across multiple organs in advanced CVD, we next studied relations of the frailty proteome to health status and disease‐free longevity. In proteins associated with any frailty axis in our AS studies that were measured in a large Icelandic cohort (70 proteins; Ferkingstad et al., 2021), we found (1) a limited effect of age on protein concentration and (2) associations between proteins and multi‐organ morbidity generally in a directionally plausible manner (Figure 5a). Of note, queried proteins were strongly related to metabolic‐inflammatory phenotypes not directly cardiac (glycemic control, body composition, inflammatory markers, malignancy).

We next studied the relation of our composite proteomic frailty axes scores in the 1894 FHS participants to frailty measures, and cause‐specific mortality. Recalibration efforts (described in Statistical methods) were excellent (Spearman *ρ* range 0.89–0.92), with resulting scores in FHS demonstrating similar sex‐based differences and limited association with age (Pearson |*r*| 0.06–0.11; Figure S8). Proteins used to recalibrate the scores from our discovery cohort (Olink) to the FHS (SomaScan) demonstrated a moderate correlation (median Spearman *ρ* = 0.58 [25%–75%: 0.21–0.71]), where available, in published data (Katz et al., 2022). Protein scores for body composition and physical function (at FHS Exam 5) exhibited generally concordant relation to visceral and subcutaneous fat or measures of physical function/frailty, respectively, at a median 6.9 years later (for frailty measures), though with mitigation of effect size after age‐ and sex‐adjustment for several measures, consistent with the broader age range in FHS (Table S4).

We next examined the relation of protein scores with cause‐specific mortality in FHS (Table 2). At a median 26 years after proteomics (25th–75th percentile 19–27 years, 755 deaths, 211 CVD‐related), a higher physical function protein score was associated with lower all‐cause mortality in FHS (Figure 5b; Table 2). Given the strong association between all‐cause mortality and the proteomics of physical function, we next sought to examine whether that mortality association would be driven by non‐cardiovascular (versus cardiovascular) causes. We carried that score forward into competing risk models for CVD versus non‐CVD mortality in FHS, where we found that the proteomics of physical function were associated with non‐CVD mortality in FHS (Figure 5b; Table 2).

**TABLE 2:** Protein scores of frailty are associated with all‐cause mortality and non‐cardiovascular mortality. Cox regression models for all‐cause mortality and Fine‐Gray competing risk models for CVD and non‐CVD mortality.

Variable	Model A^a^		Model B^b^	
	HR (95% CI)	*p* value	HR (95% CI)	*p* value
All‐cause mortality (*N* = 1894; deaths = 755)				
Protein score (PC3)	0.82 (0.76, 0.88)	1.23e‐07	0.85 (0.78, 0.92)	3.47e‐05
Protein score (PC2)	1.04 (0.96, 1.13)	0.36	0.98 (0.90, 1.08)	0.71
Protein score (PC1)	0.88 (0.81, 0.95)	9.68e‐04	0.91 (0.84, 0.99)	0.02
Fine‐Gray competing risk model (*N* = 1890; CVD deaths = 211; non‐CVD deaths = 544)				
Protein score (PC3)—CVD death	0.95 (0.82,1.1)	0.52	1.07 (0.91,1.25)	0.41
Protein score (PC3)—Non‐CVD death	0.83 (0.75, 0.91)	5.4e‐05	0.83 (0.75, 0.91)	1.2e‐04

^a^ Adjusted for sex and age.

^b^ Adjusted for sex, age, BMI, smoking status, diabetes, anti‐hypertensive medication treatment, total cholesterol/HDL cholesterol, systolic blood pressure, and prevalent CVD.

## 3 DISCUSSION

Here, we quantify 979 circulating proteins in 809 older individuals with severe AS to identify a proteomic “fingerprint” of frailty defined across 12 measures (Afilalo et al., 2017) spanning physical function, cognition, nutrition, biochemistry, self‐reported well‐being, and body composition. We determined proteomic correlates of frailty measures, specifying canonical pathways of organ function (e.g., inflammation, cell growth and senescence, cachexia) as well a host of mediators of tissue‐specific biology not previously widely reported in human frailty (e.g., myogenesis, adipose tissue inflammation, and lysosomal metabolism). Protein scores of three major frailty axes defined by integrating 12 frailty measures and proteins were strongly related to mortality after cardiac intervention, independent of clinical risk. Despite reported statistically significant age association in epidemiologic cohorts with a broader range of age (Ferkingstad et al., 2021), BMI and sex accounted for significantly greater variability in protein scores of frailty axes than age in older patients with AS (Figure 4a). Across eight decades of life (≈20–100 years), we observed heterogeneous patterns of abundance of frailty‐related proteins across age (Figure 4c), with patterns well‐established by advanced age. Across a large number of individuals, frailty‐related proteins were associated with a broad array of non‐cardiac comorbidities and outcomes, including directionally consistent associations with mortality in long‐term follow‐up in thousands of community‐dwelling individuals (Figure 5). In FHS, we observed a significant association between the protein score corresponding to physical function with all‐cause and non‐CVD mortality over two decades. Collectively, these findings extend the growing aging literature toward the cardiovascular space and emphasize the potential for proteomic studies in the context of advanced CVD to identify functional, prognostic pathways of risk for interrogation in advanced heart disease.

Separating “biological” from “chronological” aging using molecular information has been the subject of a large body of work in aging research (Ahadi et al., 2020; Basisty et al., 2020; Emilsson et al., 2018; Lehallier et al., 2019, 2020; Sebastiani et al., 2021; Tanaka et al., 2018). Approaches that generate molecular “clocks” using epigenetic (Horvath, 2013), transcriptional (Peters et al., 2015; Shavlakadze et al., 2019), genomic (Singh et al., 2019), proteomic (Tanaka et al., 2018), and metabolomic (Cheng et al., 2015) information have been advanced to identify relevant pathways of and individuals with “accelerated” aging ultimately connected to longevity, including some reports of cause‐specific mortality (Eiriksdottir et al., 2021). While these studies have illuminated mechanisms and biomarkers of aging, most do not study individuals with CVD at older ages, where varying degrees of multi‐organ frailty (beyond chronologic aging itself) may play a critical role (Collard et al., 2012). Given the prognostic relevance and reversibility of frailty (Chang et al., 2004; Guralnik et al., 1994, 1995; Pandey et al., 2023; Perera et al., 2006; Puthoff, 2008; Volpato et al., 2008), clinical studies and care in advanced heart disease have recently prioritized frailty to optimize outcome after cardiac intervention (Afilalo et al., 2017; Denfeld et al., 2017; Flint et al., 2012; Murali‐Krishnan et al., 2015; Patel et al., 2018). Nevertheless, varied definitions across studies, difficulties in standardizing measures (e.g., grip strength Cooper et al., 2021), and lack of specificity of common metrics (e.g., grip strength, walk speed, and albumin) for specific biology challenges clinical application and mechanistic discovery outside of controlled, non‐clinical cohort studies (Kameda et al., 2020; Landino et al., 2021; Lehallier et al., 2019; Liu et al., 2022; Pan et al., 2021; Rizza et al., 2014; Santos‐Lozano et al., 2020; Sathyan et al., 2020; Walston et al., 2002). With an aging population at high‐risk for advanced heart disease eligible for high‐risk intervention (e.g., destination left ventricular assist device Flint et al., 2012), objective measures that personalize variations in clinical status and biology across individuals are critical.

Our study directly addresses these limitations by employing molecular discovery in a common clinical situation where frailty is routinely considered and prognostic (AS) (Kiani et al., 2020). Unlike prior cohort‐based reports (Liu et al., 2022; Sathyan et al., 2020; Walston et al., 2002), our cohort had a dramatically higher rate of CVD and diabetes (≈70% and ≈40% overall, respectively), consistent with CVD estimates in this age range seen clinically (Yazdanyar & Newman, 2009). In this context, the use of common frailty measures extending prognostic multi‐organ structure–function (the “Essential Frailty Toolset”; Afilalo et al., 2017) to guide discovery is a fundamental strength to move beyond clinical gestalt in frailty assessment (Ijaz et al., 2022). Furthermore, the inability of age to capture a large variation in the frailty proteome at the time of TAVI (relative to comorbidity) in our older population highlights importance of discovery in a clinical CVD context. Despite statistical age associations in up to 80% of the quantified proteome in a large age range in epidemiology, the reported effect sizes are small (Ferkingstad et al., 2021). We observed similarly weak relations with age when examining proteins related to frailty in our analysis (Figure 5a), suggesting that mechanisms beyond chronological age are likely involved in the biology of frailty. In an older population undergoing clinical cardiac intervention, it is possible that an age‐related alteration in the proteome may already be prevalent/established, with inter‐individual differences determined by comorbidity (Figure 4). This notion broadly underscores the potential importance of patient‐level heterogeneity and human molecular studies to prioritize targets for therapeutic or mechanistic discovery in frailty. Indeed, “anti‐aging” pharmacology directed at metabolism may impact the proteome decades earlier to “prepare” organs for intervention (metformin and GDF‐15; Coll et al., 2020); SGLT2 inhibition and PLIN1 (Yang et al., 2021), RNA therapeutics (Fitzgerald et al., 2017; Solomon et al., 2019).

Biologically, our results implicated broad pathways relevant to both cardiac and non‐cardiac physiology in aging around a theme of host inflammatory response, cell growth and senescence, and cachexia. Several proteins related to body composition and muscle function specified known pathways (leptin signaling, IGFBPs, GDF‐15, IL6, MSTN), concordant with prior human observations and canonical mechanisms of human frailty. For example, our results are broadly consistent with a reported fall in myostatin (MSTN) with age, a relation to greater lean mass and grip strength (in men) (Bergen III et al., 2015), and a decreased muscle oxidative capacity and force generation in MSTN‐null mice (Amthor et al., 2007). Moreover, our integrative approach facilitated discovery of an array of molecules with novel, emerging roles across a broad tissue biology relevant to aging, including adipose tissue metabolism and inflammation (e.g., PLIN1, Kern et al., 2004), activin signaling (Carlsson et al., 2009; Goebel et al., 2022), and PTX3 (Kocyigit et al., 2014), islet cell function (ADGRG1 Duner et al., 2016), muscle cell physiology (ITGA11 Grassot et al., 2014), EFNA1 (Alonso‐Martin et al., 2016), LRRN1 (McKellar et al., 2021), lysosomal metabolism (CTSL Wu et al., 2011), extracellular matrix handling and fibrosis (SDC1 Yang & Friedl, 2016), among others that specify frailty mechanisms not necessarily specific to the heart. These broad mechanistic implications are consistent with our phenotype and outcome associations across thousands of individuals in Iceland and FHS for a broad array of metabolic‐inflammatory conditions (Figure 5a) and non‐cardiovascular death (Figure 5b) that are neither fully nor directly reversible with cardiac‐only intervention. These results are consistent with all‐cause mortality in a subset of the Icelandic population across a broader age range (≈22,000 individuals, ≈20–100 years old), where several proteins related to lower physical function in our study (both canonical, e.g., GDF‐15, and more novel, e.g., MZB1, ASGR1) were associated with increased mortality (Eiriksdottir et al., 2021).

From a clinical perspective, these results are compelling given recent reports suggesting potentially greater benefit to physical rehabilitation interventions in individuals with advanced heart disease who display greater frailty (Pandey et al., 2023). The novelty of this approach is the application of broad molecular characterization to frailty at the point of its clinical utility for CVD, addressing heterogeneity in how frailty is assessed in clinical practice (Cooper et al., 2021). Certainly, direct clinical application of proteomics as an actionable biomarker requires demonstration of its reversibility with intervention and advancing from a broad “omic” space with *relative* quantification (as done in nearly all molecular studies of aging and frailty) to a more precise, select panel with *absolute* quantification. Importantly, for some therapies where earlier application in patients with more advanced multimorbidity is currently standard (e.g., TAVI), proteomics cannot be viewed as a “gatekeeper” to intervention, but as a barometer for rapid stratification of individuals in need of more aggressive pharmacologic or rehabilitative therapy (in addition to TAVI) to limit poor outcome (Kitzman et al., 2021). Indeed, our results suggest high residual risk *post*‐TAVI captured by the frailty proteome—a unique opportunity to intervene more aggressively after acute CVD has been addressed. By analogy, application of these results to interventions with high morbidity and resource utilization (e.g., ventricular assist and transplant) may offer additional *pre*‐intervention opportunities to target individuals at high risk for adjunctive intervention. While not clinically available, some proteins identified in our analysis have published pharmacologic modifiers (He et al., 2021; Vandeghinste et al., 2018). While our results suggest both cardiac and non‐cardiac implications of the frailty proteome in large populations (FHS), utilization of these or similar signatures to parse cardiac from non‐cardiac morbidity after “correction” of cardiac output deficits (e.g., with ventricular assist) is a striking potential for future work. Finally, as our proteomic platforms broaden, these clinical opportunities may be met by potential molecular targets for intervention to improve frailty, similar to what has been attempted in other spaces in heart disease (e.g., RNA‐based therapies Fitzgerald et al., 2017; Solomon et al., 2019).

Several limitations of our study merit comment. Our AS sample included several non‐continuous, non‐normal exposures and some differences in covariates between discovery and validation subsamples (Table 1), potentially biasing discovery. In particular, the validation sample is biased to include individuals with greater BMI and diabetes due to CT based measures of adiposity being unavailable in participants at the extremes of waist circumference. In addition, while PCA‐based frailty axes were internally consistent with our clinical experience and were related to outcome, a larger sample size will likely be needed to examine potential sex differences in how the proteome relates to the frailty phenome. We recognize that matching frailty‐related proteins from our AS cohort to other studies with aptamer‐based proteomics offers unique challenges, including differences in specificity profile (Katz et al., 2022). While increased variance due to SomaScan‐Olink platform differences may lead to null association (due to poor concordance for some proteins; Katz et al., 2022), we observed a consistent association with outcomes and phenotypes as well as a reasonable degree of correlation (median Spearman *r* = 0.58) between the two proteomic platforms on previously published data (Katz et al., 2022), where available, with a caveat that some proteins were negatively correlated. While the cross‐sectional relation of protein scores with frailty measures in FHS may be limited by survival bias (proteins measured ≈7 years prior to frailty measures), the longitudinal Cox regression results replicated, suggesting these protein scores capture meaningful clinical outcomes. Lack of racial diversity across samples included here is a significant limitation and reflects the limited diversity in transcatheter registries (Alkhouli et al., 2019). The use of molecular features in blood testing may facilitate broader implementation of objective measures of frailty to address racial disparities (Usher et al., 2021). Ultimately, broader studies across race, frailty, and disease states with an eventual aim of absolute quantification of risk will be essential to personalize frailty assessment rapidly with prognostic and potential mechanistic implications.

In conclusion, we offer a paradigm to move past chronological age to biological markers of frailty that are related to, but not dependent on, age. The circulating human proteome captures variability in frailty traits weakly related to age, exhibiting broad relations with metabolic‐inflammatory phenotypes, outcomes, and mechanisms not specific to the heart. Sex and BMI accounted for a larger proportion of variability in the frailty proteome relative to age itself, with studies across a wider age range suggesting that proteins relevant to post‐cardiac intervention outcomes may already be established by older age. Across younger populations at lower risk, proteomic signatures of frailty were associated with mortality, including cardiovascular and non‐cardiovascular mortality. These results underscore the importance of human proteomic studies to guide discovery of functional biomarkers and potentially pharmacologically reversible pathways to optimize early intervention and post‐intervention clinical risk in advanced cardiovascular disease. Future studies should prioritize investigating the modifiability of the frailty proteome and its correlation with mortality to establish proteomics as a potential cross‐sectional and modifiable longitudinal measure of frailty.

## 4 METHODS

**FIGURE 1:**
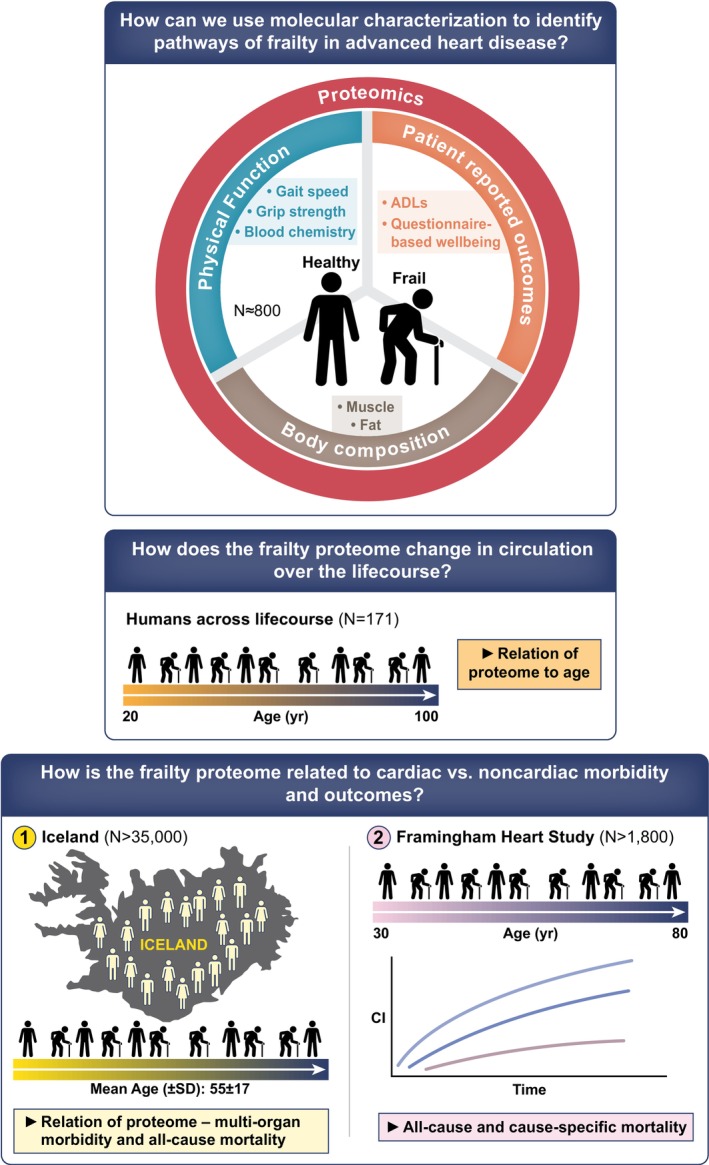
Graphical abstract and study diagram.

An overview of study design and statistical methods is shown in Figure 1 and Figure S1.

**FIGURE 2:**
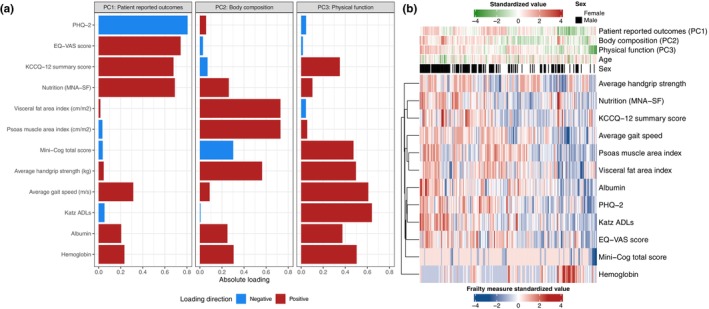
PCA of 12 frailty measures identifies 3 composite axes of frailty. (a) Loadings of the three composite axes of frailty using PCA with varimax rotation. (b) Heatmap of study participants in the derivation sample (columns) demonstrates heterogeneity in PC scores and individual measures of frailty.

**FIGURE 3:**
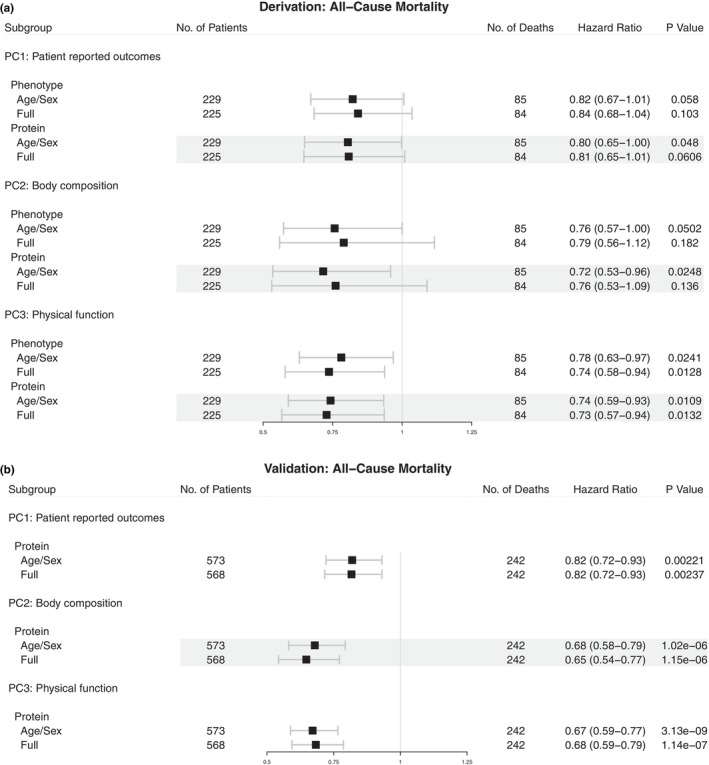
Protein‐based scores are independently related to all‐cause mortality. Forest plots of Cox regression for all‐cause mortality using phenotype and protein‐based scores in derivation (a) and validation (b) samples. Hazard ratio is expressed per 1 standard deviation increase in score. Full adjustment includes age, sex, body mass index, smoking history, diabetes, coronary artery disease, and eGFR.

**FIGURE 4:**
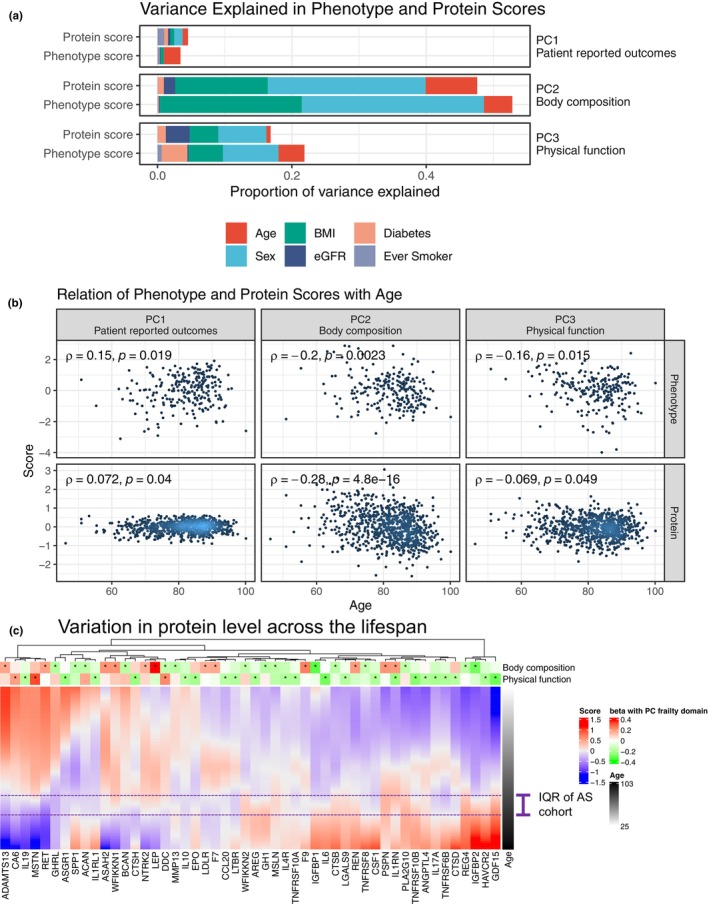
Proteomics of frailty are weakly related to age and appear to manifest decades prior to advanced age. (a) Stacked bar plot of the proportion variance explained in phenotype (frailty axes scores) and protein scores by age, sex, BMI, eGFR, diabetes, and smoking history (residuals not shown). Models for explanatory variance for phenotype scores are from the AS derivation sample with complete data on the 12 frailty measures (*N* = 233); models for protein scores pool all 809 participants (AS derivation and AS validation samples). (b) Scatterplots demonstrate relation between phenotype or protein scores with age, with correlation (Spearman). (c) Age‐related changes in plasma proteins modeled by loess (based on *Z*‐scores of protein levels) from 171 individuals (age range: 21–107 years; Lehallier et al., 2019). Proteins were selected based on association (FDR <0.10) with one of the frailty axes in the AS cohort and availability in the *N* = 171 sample. Of note, no proteins were associated with patient‐reported outcomes (PC1) in linear models (see File S1), so this is not shown in the heat bar at the top of the heatmap. The 25th–75th percentile of age in the AS cohort is shown in purple beside the age heat bar, suggesting any age‐related changes in the proteome may already be established by the time of AS intervention. *Proteins/genes with FDR <0.10 in linear models of frailty axes in the human AS cohort.

**FIGURE 5:**
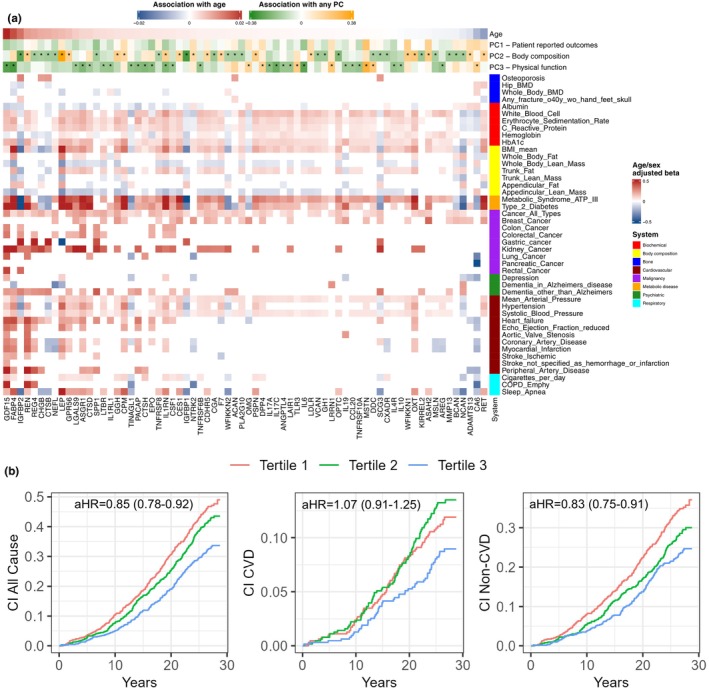
The frailty proteome, systemic multimorbidity, and cause‐specific mortality. (a) Heatmap of proteins associated with any frailty axis (FDR <0.10) also measured in >35,000 Icelanders (Icelandic Cancer Project and deCODE). Fill values are from age and sex linear adjusted models for each phenotype/outcome. The annotation bar presents the protein's relation with age in Icelanders and the protein's relation with frailty axes. Phenotype names are as provided by the parent study investigators (Ferkingstad et al., 2021). (b) Cumulative incidence curves for all‐cause mortality, cardiovascular mortality, and non‐cardiovascular mortality stratified by tertiles of protein score of physical function in FHS. These are for visualization of the survival association (unadjusted). The adjusted hazard ratio for a continuous marker (from Cox regression) is reported.

### 4.1 Study population

The discovery cohort comes from a multicenter, prospective cohort study of participants with symptomatic, severe AS undergoing TAVI (Perry et al., 2022; Stein et al., 2022). A key strength of this cohort is the systematic, prospective assessment of frailty (defined below). Severe AS was defined according to American Society of Echocardiography guidelines (peak velocity ≥4 m/s, mean gradient ≥40 mm Hg, or indexed aortic valve area <0.6 cm^2^/m^2^; Baumgartner et al., 2017). All participants in the cohort underwent TAVI. Participants were enrolled from 10 centers across the United States between May 2014 and February 2017, with a final assessment of all‐cause mortality between March and June 2020. We excluded 114 of 923 participants for not having protein data. Participants without missing data on the 12 frailty measures (“complete cases”) were included in the derivation sample, and participants with missing frailty data were evaluated for validation and prospective association. Coronary artery disease was defined as atherosclerosis in ≥1 coronary artery, prior myocardial infarction, or prior revascularization. Diabetes was defined by a participant having been diagnosed or treated for diabetes by a healthcare provider.

To contextualize and validate findings, we sought to replicate our findings using published data from multiple human cohorts. To examine age‐related changes in the frailty proteome, we analyzed data from (1) 171 individuals across the lifespan (age 21–107 years) with previously reported plasma proteomics (aptamer‐based assay, SomaScan, Somalogic) from several U.S. and European cohort studies of aging and age‐related disease (VASeattle, PRIN06, PRIN09, and GEHA; Jha et al., 2017); (2) reported cross‐sectional associations of a plasma proteome (aptamer‐based, SomaScan) with 373 phenotypes from two Icelandic cohorts (Iceland Cancer Project and deCODE genetics) comprised of 35,559 community‐dwelling adults with and without cancer (Ferkingstad et al., 2021); (3) participants from the FHS Offspring cohort with prospective frailty phenotypes and cardiovascular and non‐cardiovascular outcomes over median 26 year follow‐up with aptamer‐based plasma proteomics (SomaScan) (Ngo et al., 2016). Cardiovascular disease in FHS was defined as prior myocardial infarction, coronary death, angina pectoris, coronary insufficiency, heart failure, stroke or transient ischemic attack, or intermittent claudication (D'Agostino Sr. et al., 2008). The Institutional Review Board at each institution approved each study.

### 4.2 Proteomic profiling

#### 4.2.1 Discovery cohort (AS/TAVI)

Venous blood was collected in AS samples prior to TAVI, processed within 30 minutes, and stored at −80°C. Plasma proteins were quantified using the Olink Explore 1536 panel (Olink, Uppsala, Sweden) in three batches (Assarsson et al., 2014). Proteins related to frailty axes in the discovery cohort were matched to proteins in the replication cohorts using UniProt identifier.

We excluded 258 proteins from the Oncology panel across all batches due to a technical issue in the first batch that limited accuracy of these proteins. We used median normalization approaches to perform batch correction (with batch 3 as the referent, given most samples were run in this batch). We excluded 154 proteins if >25% of reported values were below the reported level of detection and excluded 145 proteins with a coefficient of variation greater than 40%, yielding 979 proteins available for analysis. Protein levels (in normalized protein expression units, log_2_ scale) were mean‐centered and standardized to unit variance for modeling.

#### 4.2.2 Replication cohorts

Aptamer‐based proteomics (SomaScan) was used in all replication studies (Ferkingstad et al., 2021; Lehallier et al., 2019; Nayor et al., 2020). Published data were used for Icelandic participants (Ferkingstad et al., 2021) and the U.S. and European cohort studies of aging and age‐related disease (VASeattle, PRIN06, PRIN09, and GEHA; Lehallier et al., 2019). For FHS, proteomics was performed in two batches as described (Nayor et al., 2020). FHS investigators accounted for batch effects as previously described (Nayor et al., 2020), via log‐transforming and standardizing proteins in each batch separately, pooling batches, and subsequently rank normalizing the entire FHS sample. Plate‐adjusted standardized residuals were subsequently used for regression to address batch effects comprehensively.

### 4.3 Frailty assessment

The discovery (AS) cohort prospectively assessed measures of frailty in all participants. Since there is not one universally accepted definition of frailty, for this analysis we selected elements of the Fried frailty phenotype combined with variables included in the Afialo toolset that was developed specifically for the TAVI population then conducted a PCA to define axes (or dimensions) of frailty. We did not include categorical measures of frailty (such as exhaustion or unintentional weight loss from the Fried frailty phenotype) due to their heavy weightings in the discovery cohort (e.g., almost all participants reporting no unintentional weight loss) and incompatibility with PCA. We included 12 separate measures of frailty including questionnaire‐based assessments, functional assessments, and biochemical and radiographic measures (Table S1). Three global assessments of frailty and quality of life were assessed via questionnaire, and included Katz Index of Independence in Activities of Daily Living (ADL) score (Katz et al., 1970), EuroQol Visual Analogue Scale (EQ‐VAS; Nancy Devlin & Janssen, 2020), and the Kansas City Cardiomyopathy Questionnaire summary score (KCCQ‐12; Green et al., 2000). Physical frailty was assessed by average handgrip strength (by dynamometer), average gait speed (5‐meter walk time), visceral fat area indexed to height^2^, and psoas muscle area indexed to height^2^. For participants who were unable to perform the gait speed test, a value of 0 was imputed. Pre‐TAVI computed tomography (CT) scans were used to measure psoas muscle area index and visceral fat area index using OsiriX software (Rosset et al., 2004). Bilateral psoas muscle area and visceral fat area were measured manually on a single 3 mm slice at the L4 level in the transverse plane. Cognitive (Mini‐Cog total score; Borson et al., 2003), psychosocial (Patient Health Questionnaire‐2 [PHQ‐2] Kroenke et al., 2003), and nutritional measures (Mini Nutritional Assessment‐Short Form [MNA‐SF]) were included as additional metrics of frailty (Rubenstein et al., 2001). Finally, we included hemoglobin and albumin given their inclusion in the Essential Frailty Toolset and association with post‐TAVI outcomes (Afilalo et al., 2017).

### 4.4 Statistical analysis

#### 4.4.1 Summarizing 12 frailty measures in composite phenotype measures

We observed correlations among measures of frailty (Figure S1), prompting an approach to generate composite axes of frailty using PCA. We conducted PCA (with varimax rotation; using *psych* in R; Revelle, 2022) on participants with complete data on all 12 measures of frailty (“derivation” sample, *N* = 233). Frailty measures were mean‐centered and standardized (mean = 0, variance = 1) for PCA. Principal components were selected by examination of a scree plot (generating 3 PCs summarizing 12 component frailty) and labeled based on loadings for that PC. Each PC score was used individually as a summary measure of its composite axis in downstream models.

#### 4.4.2 Identifying proteomic correlates of frailty

To identify proteins related to frailty within the derivation sample, we used linear regression with individual proteins as independent variables and each of the 12 measures of frailty as dependent variables, with adjustments for age and sex. A false discovery rate (FDR; Benjamini–Hochberg method) was used to control type 1 error. This regression approach was repeated with frailty axes scores (from PCA) as dependent variables. Proteins associated with the 12 measures of frailty (FDR <0.05) were selected and mapped to both KEGG pathways and the Reactome database (clusterProfiler in R; Wu et al., 2021) respectively. Given our proteomic coverage did not cover all circulating proteins (*N* = 979), we used statistical tests only to select pathways for visualization (*p* values were generated by hypergeometric tests and adjusted by Benjamini–Hochberg method). Proteins associated with any of the three frailty axes (generated by PCA) with an FDR <0.10 were examined in external datasets.

To construct parsimonious models for frailty, we next used LASSO regression (*caret* in R Kuhn, 2008) with frailty axes scores as dependent variables and all proteins (standardized to mean = 0, variance = 1) as penalized independent variables. LASSO models were developed in the subset of participants in the discovery (AS) cohort included in the PCA (derivation sample as mentioned above). Cross‐validation (10 folds, with 5 repeats) was used to optimize model hyperparameters (e.g., lambda). Resulting models were then used to create protein scores for each of the frailty PCs, by taking the sum of the product of each regression coefficient and protein level for each individual. These protein scores represent a blood‐based proteomic “fingerprint” of frailty for downstream analyses. To understand the proportion of variance explained by age and morbidity for each score, we performed type I ANOVA in models for each proteomic and phenotype score as a function of age, sex, body mass index (BMI), diabetes, smoking history, and renal function (estimated glomerular filtration rate [eGFR] by the CKD‐EPI equation; Miller et al., 2022).

To validate these proteins scores of frailty, we imputed missing phenotype data in the 576 validation sample participants (individuals with AS without complete cases for 12 measures of frailty), using multivariate imputation by chained equations (R package *mice*; van Buuren & Groothuis‐Oudshoorn, 2011). We applied the PCA model from the derivation sample in the validation sample (with imputed data) and correlated the resulting composite PC‐based phenotypes with the protein scores from LASSO. While we recognize that the CT measures may not be fully missing at random (potentially limiting imputation accuracy), given that this cohort was a highly unique set with severe cardiac disease (AS), proteomics, and follow‐up, we conducted this analysis to test the generalizability of our result in the derivation sample. The imputed data were not used in any other part of the analysis.

#### 4.4.3 Association of frailty with mortality in symptomatic, severe AS

We entered frailty axes scores or their proteomic surrogates (by LASSO) as independent variables in Cox regression for all‐cause mortality. Of note, our AS sample did not have cause‐specific mortality data reported. These regressions were performed in our derivation sample (*N* = 233), as well as a validation sample (*N* = 576). Models were adjusted for age, sex, BMI, smoking history, diabetes, coronary artery disease, and eGFR. In a sensitivity analysis, we further adjusted for hemoglobin, albumin, and NT‐proBNP to address potential confounding.

#### 4.4.4 Studies in replication cohorts

We utilized several published studies (see Section 4.1) to further characterize relations of age and morbidity with circulating proteins corresponding to the “frailty proteome” identified in the AS cohort. All proteins associated with any frailty axis with an FDR <0.10 (relaxed to allow maximal discovery across the derivation AS sample and the replication cohorts) were selected and mapped to their published data by UniProt identifiers. In a cross‐sectional study of 171 individuals across the lifespan (age 21–107 years; Lehallier et al., 2019), we used LOESS models to describe the relation between protein level and age, with visualization of age‐related predicted trajectories from these models. We leveraged a study of 35,559 community‐dwelling adults in Iceland (Ferkingstad et al., 2021) to examine the relation between proteins (those significantly associated with any frailty axis also present in the Iceland dataset, matched by UniProt) with (1) age (from reported linear regression for protein as a function of age and sex) and (2) a subset of 44 reported phenotypes selected from a total 373 phenotypes based on relevance to aging across multiple systems (linear/logistic regression for phenotype as a function of protein, age, and sex).

#### 4.4.5 Framingham Heart Study

We examined the association of protein scores with frailty measures and long‐term CVD and non‐CVD outcomes in the FHS Offspring cohort. We used recursive feature elimination on 367 proteins common to FHS and the AS cohort (determined by matching on UniProt identifier) in linear models (in *caret* Kuhn, 2008, with a 5% tolerance) to recalibrate scores developed using Olink data in our discovery sample (AS) for FHS (given the differences in proteomic coverage). Recalibration fit using this approach was good (Spearman *ρ* range = 0.89–0.92 across the 3 scores). The refitted models were then applied to FHS by summing the product of each regression coefficient (from recursive feature elimination) and protein level for each individual. We used logistic and linear regression to relate protein scores (from FHS Exam 5) to frailty measures collected at FHS Exam 7 (≈7 years later), including Rosow–Breslau questions (ability to do heavy work, ability to walk a half mile), Katz ADLs, grip strength (Jamar dynamometer), gait speed (4‐meter walk at usual pace), and time to complete five chair stands (Liu et al., 2016). In addition, we included relations with visceral and subcutaneous adiposity as a measure of body composition (Fox et al., 2010). We used Cox regression to relate each of the 3 protein scores with all‐cause mortality with adjustments for sex and age in minimally adjusted models, with further adjustments for BMI, smoking status, diabetes, anti‐hypertensive medication treatment, total and HDL cholesterol, systolic blood pressure, and prevalent CVD. We then used a competing risk model (Fine–Gray) to evaluate for CVD versus non‐CVD mortality for protein scores that were associated with all‐cause mortality in standard Cox models (D'Agostino et al., 2008; Fine & Gray, 1999).

R (versions 4.2.1 and 4.2.2) was used for analyses. A two‐tailed *p* value less than 0.05 (with type 1 error control as specified above) was considered statistically significant.

## AUTHOR CONTRIBUTIONS

Matthew Nayor, Sammy Elmariah, Brian R. Lindman, and Ravi Shah directed and supervised this study. Andrew S. Perry, Matthew Nayor, Sammy Elmariah, Brian R. Lindman, and Ravi Shah conceptualized and designed the study. Benoit Lehallier, Sangeeta Nair, Colin Neill, J. Jeffrey Carr, William Fearon, Samir Kapadia, Dharam Kumbhani, Linda Gillam, Laurie Farrell, Robert E. Gerszten, Sammy Elmariah, and Brian R. Lindman contributed to the acquisition of data. Andrew S. Perry, Shilin Zhao, Priya Gajjar, and Patricia Miller performed the statistical analysis. All authors critically reviewed and revised the manuscript, and all authors approved the final version.

## FUNDING INFORMATION

A.S.P. is supported by grants from the NIH and American Heart Association Strategically Focused Research Network in Cardiometabolic Disease. R.S. and V.L.M. are supported by research grants from the NIH and American Heart Association Strategically Focused Research Network in Cardiometabolic Disease. M.M.M. is supported by NIH/NIA 5K01AG075143. B.R.L. is supported by R01AG073633.

## CONFLICT OF INTEREST STATEMENT

A.S.P. is supported in part by grants from the NIH and American Heart Association. V.L.M. has received grant support from Siemens Healthineers, NIDDK, NIA, NHLBI, and AHA. He has received other research support from NIVA Medical Imaging Solutions. He owns stock in Eli Lilly, Johnson & Johnson, Merck, Bristo‐Myers Squibb, Pfizer, and stock options in Ionetix. He has received research grants and speaking honoraria from Quart Medical. B.L. is full time employee of Alkahest Inc. W.F. receives institutional research support from Edwards, Abbott, Boston Scientific, and Medtronic. L.G. is an advisor to Bracco Diagnostics, Philips, and Edwards Lifesciences and directs an imaging core laboratory with contracts with Edwards Lifesciences, Medtronic, and Abbott (no direct compensation). S.E. has received research grants from Edwards Lifesciences, Medtronic, and Abbott and consulting fees from Edwards Lifesciences. M.N. received speaking honoraria from Cytokinetics. B.R.L. has served on the scientific advisory board for Roche Diagnostics and has received research grants from the NIH, Edwards Lifesciences, and Roche Diagnostics. R.S. is supported by grants from the NHLBI, NIA, and NIDDK and has served as a consultant for Amgen, Cytokinetics, Myokardia, and Best Doctors. He is a co‐inventor on a patent for ex‐RNAs signatures of cardiac remodeling.

## Supporting information


Data S1.

## Data Availability

Data from the AS discovery cohort are available from the corresponding author on reasonable request. Data from replication cohorts are publicly available (Ferkingstad et al., 2021; Lehallier et al., 2019), including FHS data (via contacting FHS, www.framinghamheartstudy.org).
