# Supplementary material for: Proteomic architecture of frailty across the spectrum of cardiovascular disease
Source: Aging Cell. 2023 Sep 20;22(11):e13978. doi: 10.1111/acel.13978 (PMC10652351; doi:10.1111/acel.13978)
Supplement: Supplementary file 1 — Data S1. [file ACEL-22-e13978-s001.zip › Supplement for proteomics frailty manuscript.docx]

**Data Supplement for** Perry et. al*, “Proteomic architecture of frailty across the spectrum of cardiovascular disease”*

**List of Enrolling Centers**

Barnes-Jewish Hospital, St. Louis, MO

Cleveland Clinic Foundation, Cleveland, OH

Dartmouth-Hitchcock Medical Center, Lebanon, NH

Intermountain Heart Institute, Murray, UT

Massachusetts General Hospital, Boston, MA

Morristown Medical Center, Morristown, NJ

Stanford Medical Center, Palo Alto, CA

University of Texas Southwestern Medical Center, Dallas, TX

University of Utah Hospital, Salt Lake City, UT

Vanderbilt University Medical Center, Nashville, TN

**Supplementary Data Tables** (Supplemental_Data.xlsx)

**SD01**: Protein dictionary.

**SD02**: Results of age/sex adjusted linear models for individual measures of frailty in the entire AS cohort (both derivation and validation samples). Proteins and outcomes were standardized. False discovery rate (FDR) method was Benajmini-Hochberg.

**SD03**: Results of age/sex adjusted linear models for composite axes (PCs) of frailty in the derivation sample (AS cohort). Proteins and outcomes for all models were standardized. False discovery rate (FDR) method was Benajmini-Hochberg.

**SD04**: Results from LASSO regression model for each composite axis of frailty (PC) as a function of all proteins. Models trained in the derivation sample (AS cohort).

**SD05**: Correlation matrices for Figure 3C reporting the Spearman rho, number of observations, and P values.

**SD06**: KEGG Pathway analysis results

**SD07**: REACTOME Pathway analysis results

**Supplemental Table 1: Frailty measures and method of assessment used in the discovery cohort**

| **Frailty Domain** | **Frailty Measure** | **Method of Assessment** |
| --- | --- | --- |
| Global | Katz ADL | Questionnaire assessing 6 domains of independence: bathing, dressing, toileting, transferring, continence, feeding. Scores range from 0-6 with 0 being very dependent and 6 being independent. |
|  | EuorQol Visual Analog Scale | Vertical scale where participants are asked to provide a general assessment of their overall health. Scores range from 0-100 with 0 being worst imaginable health and 100 being best imaginable health. |
|  | Kansas City Cardiomyopathy Questionnaire | Questionnaire assessing 4 domains of health status: symptoms, physical function, quality of life, social limitation. Scores range from 0-100 with 0 being very poor health status and 100 excellent health status |
| Physical | Handgrip | Dynamometer |
|  | Gait speed | 5-meter walk |
|  | Visceral fat area | CT scan |
|  | Psoas muscle area | CT scan |
| Cognitive | Mini-Cog score | Neurocognitive testing involving recall and clock drawing. Scores of 0-2 suggest higher likelihood of dementia. Scores of 3-5 suggest lower likelihood of dementia |
| Psychosocial | Patient Health Questionnaire-2 | Questionnaire with 2 questions used as a screening tool for depression. Scores range from 0-6 with 0-2 having a low likelihood of depression and >3 having a high likelihood of depression. |
| Nutrition | Mini Nutritional Assessment – Short Form | Questionnaire used to screen for malnutrition. Scores range from 0-14, with 0-7 being malnourished and 12-14 being normal nutritional status. |
| Biochemical | Hemoglobin | Venous blood draw |
|  | Albumin | Venous blood draw |

**Supplemental Table 2: FHS baseline characteristics.** Continuous variables are reported as mean ± standard deviation (SD). Categorical variables are reported as N (%).

| **Characteristic** | **N** | **Value** |
| --- | --- | --- |
| Age, years | 1894 | 55.0 ± 10.0 |
| Women | 1894 | 1,013.0 (53.5%) |
| Non-white | 1894 | 22.0 (1.2%) |
| Current smoker | 1894 | 369.0 (19.5%) |
| Body mass index (kg/m^2^) | 1894 | 27.4 ± 5.1 |
| Diabetes | 1894 | 149.0 (7.9%) |
| Creatinine (mg/dL) | 1606 | 1.0 ± 0.2 |
| Hypertension treatment | 1894 | 370.0 (19.5%) |
| Total cholesterol (mg/dL) | 1894 | 204.5 ± 36.2 |
| Hight density lipoprotein (mg/dL) | 1894 | 50.0 ± 15.1 |
| Systolic blood pressure (mmHg) | 1894 | 126.4 ± 19.0 |
| Prevalent CVD | 1894 | 117.0 (6.2%) |
| Grip strength (kg) | 1062 | 32.7 $\pm13.1$ |
| Gait speed (m/s) | 1062 | 3.5 $\pm$1.1 |
| Time to complete 5 chair stands (seconds) | 1062 | 11.9 $\pm$3.4 |
| “Independent” by Katz ADL | 1626 | 1493 (91.8) |
| Rosow-Breslau: Ability to do heavy work | 1626 | 1481 (91.1) |
| Rosow-Breslau: Ability to walk half-mile | 1626 | 1524 (93.7) |
| Visceral fat volume (cm^3^) | 686 | 2,130.7 ± 1,101.0 |
| Subcutaneous fat volume (cm^3^) | 686 | 2,984.4 ± 1,320.5 |

**Supplemental Table 3: Cox model results for sensitivity analysis in aortic stenosis validation sample (adjusted for age, sex, BMI, diabetes, CAD, smoking, eGFR, hemoglobin, albumin, NTproBNP*)**

| **PC** | **HR (95% CI)** | **P Value** | **N** | **Deaths** |
| --- | --- | --- | --- | --- |
| PC1 | 0.94 (0.80-1.09) | 0.396 | 552 | 235 |
| PC2 | 0.71 (0.58-0.87) | 7.95e-04 | 552 | 235 |
| PC3 | 0.76 (0.65-0.90) | 0.001 | 552 | 235 |

*NTproBNP was log-transformed prior to use in Cox regression.

**Supplemental Table 4: Relation of protein scores to physical function and frailty metrics in the Framingham Heart Study.**

**A.** Odds ratios between protein scores at exam 5 and Rosow Breslau questions at Exam 7 (N=1626)

|  |  | **Unadjusted** | | **Adjusted for age and sex** | |
| --- | --- | --- | --- | --- | --- |
| **Outcome** | **Protein score** | **OR (95% CI)** | **P Value** | **OR (95% CI)** | **P Value** |
| Heavy work^a^ | PC1 | 1.26 (1.06, 1.50) | 8.58e-03 | 1.10 (0.91, 1.33) | 0.32 |
|  | PC2 | 1.25 (1.06, 1.48) | 9.79e-03 | 1.00 (0.82, 1.22) | 0.97 |
|  | PC3 | 1.52 (1.28, 1.81) | 1.95e-06 | 1.38 (1.14, 1.66) | 6.94e-04 |
| Half-mile^b^ | PC1 | 1.38 (1.13, 1.69) | 1.95e-03 | 1.28 (1.02, 1.60) | 3.14e-02 |
|  | PC2 | 1.11 (0.91, 1.35) | 0.31 | 0.88 (0.70, 1.12) | 0.3 |
|  | PC3 | 1.28 (1.04, 1.56) | 1.72e-02 | 1.14 (0.92, 1.42) | 0.22 |
| Katz ADL (Independent vs. not | PC1 | 1.38 (1.15, 1.66) | 4.31e-04 | 1.18 (0.97, 1.44) | 0.11 |
|  | PC2 | 1.21 (1.01, 1.44) | 3.67e-02 | 0.85 (0.69, 1.05) | 0.15 |
|  | PC3 | 1.30 (1.09, 1.56) | 3.47e-03 | 1.10 (0.91, 1.34) | 0.33 |

^a^Rosow-Breslau: Are you able to do heavy work around the house, like shovel snow or wash windows, walls, or floors without help.

^b^Rosow-Breslau: Are you able to walk half a mile without help (about 4 - 6 blocks).

**B.** Cross-sectional associations between protein scores at exam 5 and grip strength, gait speed, and chair stands at exam 7 (N=1062).

|  |  | **Unadjusted** | | **Adjusted for age and sex** | |
| --- | --- | --- | --- | --- | --- |
| **Outcome** | **Protein score** | $\boldsymbol{\beta\pm}$ **SE** | **P Value** | $\boldsymbol{\beta\pm}$ **SE** | **P Value** |
| Grip strength | PC1 | 0.31 ± 0.03 | 2.12e-25 | 0.01 ± 0.02 | 0.55 |
|  | PC2 | 0.41 ± 0.03 | 4.96e-45 | 0.00 ± 0.02 | 0.88 |
|  | PC3 | 0.30 ± 0.03 | 6.09e-24 | 0.06 ± 0.02 | 0.003 |
| Gait speed | PC1 | -0.06 ± 0.03 | 0.05 | -0.02 ± 0.03 | 0.51 |
|  | PC2 | 0.00 ± 0.03 | 0.98 | 0.08 ± 0.04 | 0.02 |
|  | PC3 | -0.07 ± 0.03 | 0.03 | -0.04 ± 0.03 | 0.24 |
| Time to complete 5 chair stands | PC1 | -0.04 ± 0.03 | 0.18 | -0.03 ± 0.03 | 0.35 |
|  | PC2 | 0.00 ± 0.03 | 0.96 | 0.03 ± 0.04 | 0.45 |
|  | PC3 | -0.10 ± 0.03 | 0.001 | -0.10 ± 0.03 | 0.002 |

Estimated beta coefficients represent the SD change in outcome for a 1-SD higher value of protein score.

**C.** Linear regression models between protein scores visceral and subcutaneous fat (N=686).

|  |  | **Unadjusted** | | **Adjusted for age and sex** | |
| --- | --- | --- | --- | --- | --- |
| **Outcome** | **Protein score** | $\boldsymbol{\beta\pm}$ **SE** | **P Value** | $\boldsymbol{\beta\pm}$ **SE** | **P Value** |
| Log visceral fat volume (cm^3^) | PC1 | 0.09 ± 0.04 | 2.6e-02 | -0.06 ± 0.04 | 0.1 |
|  | PC2 | 0.47 ± 0.03 | 2.05e-38 | 0.37 ± 0.04 | 9.85e-23 |
|  | PC3 | -0.02 ± 0.04 | 0.61 | -0.15 ± 0.04 | 1.45e-05 |
| Log subcutaneous fat volume (cm^3^) | PC1 | -0.07 ± 0.04 | 0.07 | 0.00 ± 0.04 | 0.91 |
|  | PC2 | 0.20 ± 0.04 | 6.91e-08 | 0.40 ± 0.04 | 3.25e-21 |
|  | PC3 | -0.19 ± 0.04 | 1.05e-06 | -0.14 ± 0.04 | 2.52e-04 |
| Log (visceral fat volume/subcutaneous fat volume) | PC1 | 0.15 ± 0.04 | 5.42e-05 | -0.06 ± 0.03 | 3.57e-02 |
|  | PC2 | 0.33 ± 0.04 | 1.97e-19 | 0.06 ± 0.03 | 0.06 |
|  | PC3 | 0.14 ± 0.04 | 2.21e-04 | -0.04 ± 0.03 | 0.14 |

Estimated beta coefficients represent the SD change in log-transformed outcome for a 1-SD higher value of protein score.

**Supplemental Figure Legend**

**Supplemental Figure 1: Analysis overview.** Created on Biorender.com.

**Supplemental Figure 2: Interrelatedness of frailty measures within the aortic stenosis cohort.** Spearman correlation heatmap demonstrates the interrelatedness of 12 frailty measures within the derivation cohort. Asterisk indicates nominal P value <0.05. ADLs = activities of daily living; EQ-VAS = EuroQol-visual analogue scales; KCCQ = Kansas City Cardiomyopathy Questionnaire; MNA-SF = Mini-Nutritional Assessment – Short Form; PHQ-2 = Patient Health Questionnaire-2.

**Supplemental Figure 3: Frailty scores by sex.** Frailty PC scores demonstrate sex-specific differences with males tending to have higher scores in PC2 and PC3 within the derivation cohort.

**Supplemental Figure 4: Model fit for LASSO based protein scores of frailty.** (A) Boxplots of the cross-validated R^2^ (in hold-out folds) for protein-based LASSO models of each frailty axis demonstrates a weak relation between protein scores and patient reported outcomes, with moderate relations between protein scores and body composition and physical function. (B) Scatterplot with Spearman correlation between protein scores and frailty axes in the derivation sample (N=233).

**Supplemental Figure 5: LASSO generated protein scores of frailty capture similar information as the parent frailty phenotype axis.** Spearman correlations between frailty scores (both phenotype and protein-based) and the 12 measures of frailty in the derivation cohort (N=233). Asterisk indicates nominal P value less than 0.05. Correlation values, number of observations and P values are reported in the **Online Supplementary Data File (SD04)**.

**Supplemental Figure 6: Pathway analysis heatmap.** Pathway analysis from KEGG (A) and REACTOME (B) databases using age/sex adjusted linear model results relating individuals proteins to individual measures of frailty. Cell fill reflects the negative log_10_ transformed enrichment P value of proteins associated with each frailty measure. Corresponding data tables are in the **Online Supplementary Data File (SD06, SD07)**.

**Supplemental Figure 7: Age-related trajectories in select plasma protein levels.** Data from 171 individuals (Lehallier et. al. *Nature Medicine* 2019; citation in text). Here we show several proteins representative of different trajectories of age-related changes. Visualization of all matched proteins is shown in **Figure 4** in the main text.

**Supplemental Figure 8: Distribution of protein scores by sex and age in FHS cohort.** (A) Histograms demonstrate similar differences by sex in protein score in FHS as was seen in the AS cohort. (B) Scatterplots demonstrate a similar lack of relation between age and protein score in FHS as was seen in the AS cohort.

**Supplemental Figure 1**

**
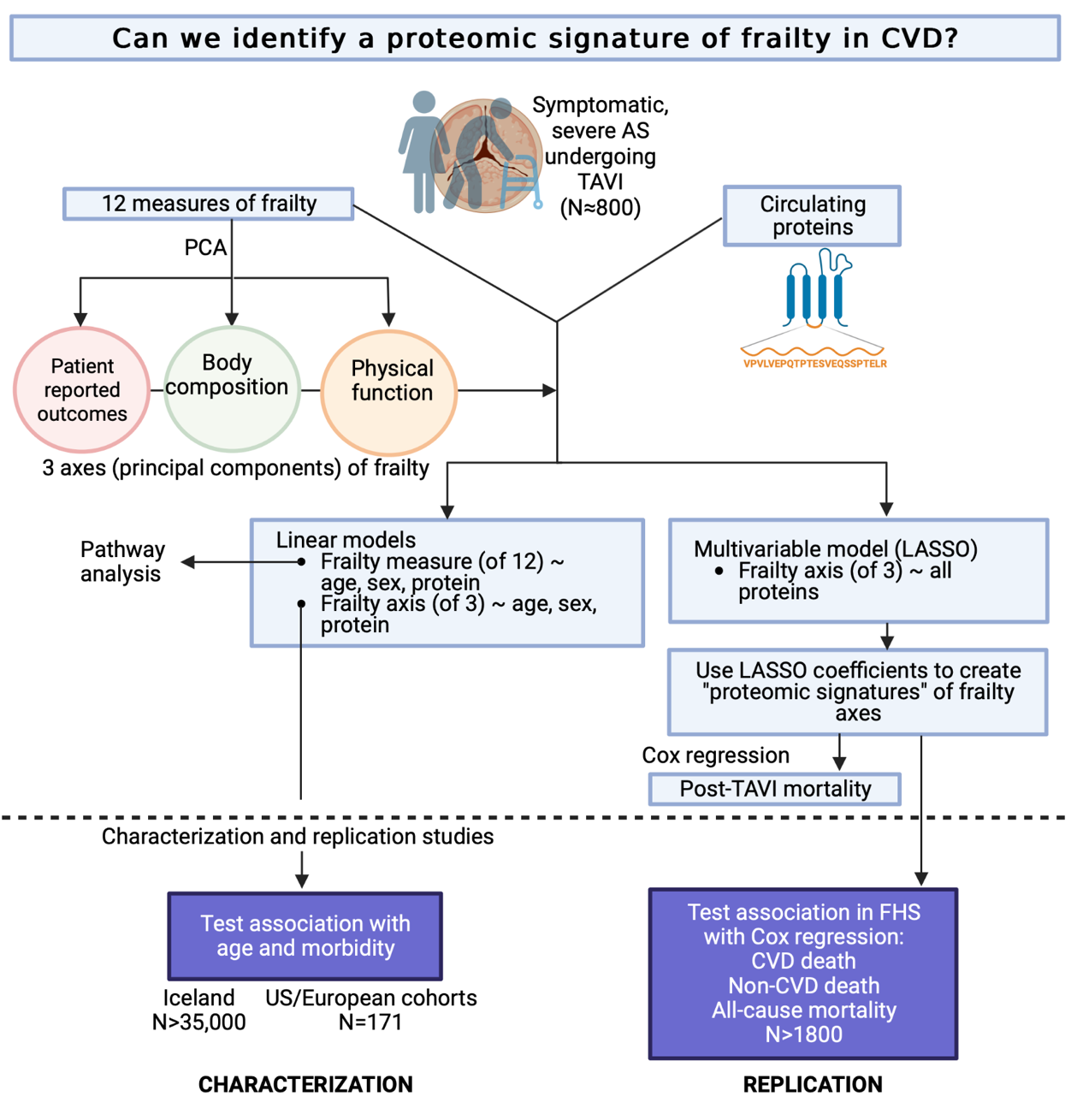
**

**Supplemental Figure 2**

**Supplemental Figure 3**

**Supplemental Figure 4**

**(A)**

**(B)**

**Supplemental Figure 5**

**Supplemental Figure 6A**

**Supplemental Figure 6B**

**Supplemental Figure 7**

**Supplemental Figure 8**
